# Perceptions and clinical practices associated with post-TB health and well-being in the Netherlands

**DOI:** 10.5588/ijtldopen.24.0377

**Published:** 2024-10-01

**Authors:** I. Spruijt, A. Verhage, F. Procee, N. Vrubleuskaya, C. Mulder, O. Akkerman

**Affiliations:** ^1^ Department TB Elimination and Health System Innovations, KNCV Tuberculosis Foundation, The Hague, The Netherlands;; ^2^ Department of Paediatric Infectious Diseases & Immunology, University of Groningen, University Medical Center Groningen, Groningen, The Netherlands;; ^3^ Department of TB Care and Control, Municipal Public Health Services Amsterdam, Amsterdam The Netherlands;; ^4^ Department of Global Health, Amsterdam University Medical Centers, University of Amsterdam, Amsterdam, The Netherlands;; ^5^ Department of TB Care and Control, Municipal Public Health Service, The Hague, The Netherlands;; ^6^ National Institute for Public Health and the Environment, Bilthoven, The Netherlands;; ^7^ Amsterdam Institute for Global Health and Development, Amsterdam University Medical Centres, Amsterdam, the Netherlands;; ^8^ Department of Pulmonary Diseases and Tuberculosis, University of Groningen, University Medical Center Groningen, Groningen, The Netherlands;; ^9^ TB Center Beatrixoord, University of Groningen, University Medical Center Groningen, Groningen, The Netherlands.

**Keywords:** tuberculosis, low-incidence country, post-TB lung disease, PTLD, quality of life

Dear Editor,

Post-TB health and well-being is a collective term for conditions that can affect the physical and mental health, as well as social and economic aspects, of TB survivors after successful completion of treatment.^1,2^ Recent studies have estimated that 18–86% of TB survivors suffer from post-TB lung disease (PTLD), a range of disorders affecting the airways, lungs and pleurae.^3^ PTLD more often occurs after delayed TB diagnosis, or extensive disease and prolonged or repeated treatments, and impedes the patient’s quality of life (QoL) and life expectancy.^3–5^ Although limited, most recent evidence comes from high TB incidence settings.^1^ Clinical experience and insights from TB survivors indicate that post-TB disease also affects TB survivors in The Netherlands (a low TB incidence setting). Current efforts in low TB incidence settings are mainly directed at maintaining TB expertise among physicians: guidelines and training activities are focused on high-quality TB diagnosis and treatment outcomes and do not include post-TB health and well-being.^6^ However, interventions for patients with PTLD have been reviewed and discussed.^7,8^ Recent clinical standards recommend evaluating patients for PTLD at the end of TB treatment, using a variety of diagnostic tests and, if lung impairment is detected, subsequent outpatient pulmonary rehabilitation.^4,9,10^ As a first step to determining the research priorities in the Netherlands, we conducted a landscape assessment focusing on knowledge and awareness of post-TB health and well-being – including PTLD. We explored current clinical practice and experiences among Dutch physicians involved in the treatment of persons with TB disease.

We conducted a cross-sectional study using a 16-item online survey using Microsoft Forms. On behalf of the authors, the Dutch Society of Pulmonology and Tuberculosis (NVALT) invited 108 pulmonologists (77 with a TB focus) and 21 TB public health physicians. Additionally, AV invited 58 paediatric infection disease specialists. A reminder was sent two months later. Data were visualised and analysed using Microsoft Excel. Participants provided consent for the use of their anonymous responses.

An overview of the responses to the survey is presented in the Table, with more detailed responses in Supplementary Data. Of 187 physicians, 40 (31%) responded to the study. Half of the physicians reported some knowledge and awareness of post-TB health and well-being. Post-TB health and well-being were expected to be a problem by 24 (60%), and most physicians estimated that less than 20% of TB survivors have PTLD and expressed the possible presence of unmet care needs among TB survivors. Current TB care in the Netherlands does not take into account post-TB health: all physicians reported follow-up actions and care for patients who show indications of persistent health complaints after completing TB treatment, such as persistent abnormalities on chest X-rays, persistent cough, complaints of dyspnoea, thoracal /other persistent pain, and general health complaints. Few physicians reported pulmonary function impairment, severe or disseminated forms of TB disease, complicated TB treatment or non-completion of TB treatment as indications for follow-up. Only one physician reported psychological issues as an indication for follow-up. Follow-up appointments are scheduled based on a patient’s needs, with timing varying from 3, 4, 6, to 12 months after completion of TB treatment, and the duration varying between 6 months, 1–2 years to an exceptional 5 years. Fifteen (38%) physicians reported they plan follow-up care with patients after TB treatment completion as the standard of care, with only a minority 6/15 (40%) performing pulmonary function tests. Considering the potential but unknown impact on the (young) patient population, a majority of physicians (27/40, 68%) indicated the importance of assessing post-TB health and well-being among TB survivors.

In practice, I see a population of mainly Turkish and Moroccan elderly with chronic pulmonary complaints who had TB in the past. We are unaware of the long-term impact of TB on my current patient population. (Pulmonologist)

Proposed research topics are type, prevalence and severity of health complaints; QoL; unmet care needs; risk factors for post-TB conditions; and potential interventions or therapies. Subsequent evidence and insights should inform guidelines and operational handbooks (Table).

We have absolutely no idea what we are looking at in the Netherlands. Worldwide, the numbers are shocking. (TB Physician)

**Table.** Responses to a survey on knowledge and awareness of post-TB health and well-being among physicians in The Netherlands.

		Total	Public health physician	Pulmonologist	Paediatrician	Infection specialist
Total		40	10	25	4	1
Q1. To what extent do you know about post-TB health and well-being	None	8	1	6	1	0
	Very little	6	1	2	3	0
	Some	20	5	14	0	1
	A lot	6	2	3	0	0

Q2. To what extent do you believe post-TB lung health and well-being is a problem among TB patients in The Netherlands?	None	2	0	2	0	0
	Very little	13	6	6	1	0
	Some	20	3	14	3	0
	A lot	5	1	3	0	1

Q3. To what extent do you believe post-TB lung disease is a problem among Dutch TB patients?	None	3	0	3	0	0
	Very little	11	6	5	0	0
	Some	24	4	16	4	0
	A lot	2	0	1	0	1

Q4. Do you follow up with all TB patients after the successful completion of TB treatment?	Yes	15	5	8	1	1
	No	25	5	17	3	0

Q5. Do you follow up with TB patients (on indication) after the successful completion of TB treatment?	Yes	40	10	25	4	1
	No	0	0	0	0	0

Q6. Do you feel there are unmet care needs among TB patients in the Netherlands? [Multiple answers]	Yes	32	10	19	2	1
	No	8	0	6	2	0

Q7. Do you think more research is warranted to assess the prevalence and severity of post-TB health and well-being and the type of interventions that should be applied in practice to improve post-TB health and well-being?	Yes	27	7	16	3	1
	No	13	3	9	1	0

CXR = chest X-ray; GP = general practitioner; COPD = chronic obstructive pulmonary disease; CT = computed tomography; MRI = magnetic resonance imaging; MDR-TB = multidrug-resistant TB.

The survey responses indicate that physicians perceive post-TB-associated disabilities as a potentially important issue that affects the health and well-being of TB survivors in the Netherlands. However, evaluation of TB patients for PTLD at the end of treatment is not (yet) part of standard care and practices and should be aligned with international clinical standards and recommendations.^8,10^ Clinical standards on post-TB recommend an evaluation of the presence of PTLD and any adverse effects due to the treatment provided at the end of TB treatment.^1,10^ The long-term sequelae from TB treatment can be identified using a series of tests, including chest imaging (e.g., fibrosis, cavities, pleural thickening, bronchiectasis, pulmonary hypertension, secondary bacterial and fungal infections); pulmonary function testing, including a 6-minute walk test, spirometry, plethysmography, diffusing capacity of the lungs for carbon monoxide, and carbon oxide transfer coefficient to detect obstructive, restrictive and mixed patterns; and cardiopulmonary exercise testing to assess the integrative responses of the cardiovascular, respiratory and musculoskeletal systems to incremental exercise in patients with PTLD. Patients with low peripheral oxygen saturation should receive arterial blood gas analysis. Furthermore, a sputum specimen for smear microscopy and culture should be obtained from adult patients with bacteriologically confirmed TB who can still produce sputum to determine treatment failure. Pulmonary and cardiopulmonary tests are only feasible in a subset of the paediatric population: lung function tests and a computed tomography scan should be considered in all children aged 4–6 years with severe TB lung involvement.^10^

TB may impact QoL long after successful completion of TB treatment. Therefore, the impact of post-TB health and well-being on the life of TB survivors should not be ignored, even in countries with relatively low levels of TB disease (e.g., in 2023, there were 710 TB patients in The Netherlands).^11^ Recommendations from clinical standards on PTLD^10^ should be incorporated into national guidelines on TB treatment. Such recommendations urgently need to be supported by high-quality evidence from low TB incidence settings on the prevalence, severity, risk factors, and treatment interventions for long-term sequelae for both children and adults.^12^ Future participatory-based mixed-methods research among children and adults should assess the consequences of TB treatment on the psycho-social and economic aspects of a TB survivor’s life. Finally, physicians caring for TB patients should be trained on current evidence and clinical recommendations regarding PTLD.

## Supplementary Material


