# Supplementary material for: Perceptions and clinical practices associated with post-TB health and well-being in the Netherlands
Source: IJTLD Open. 2024 Oct 1;1(10):480–2. doi: 10.5588/ijtldopen.24.0377 (PMC11467848; doi:10.5588/ijtldopen.24.0377)
Supplement: Supplementary file 1 [file ijtldopen24-0377_SupplementaryData1.docx]

**Perceptions and clinical practices associated with post-TB health and well-being in the Netherlands**

Supplement 1
Extended Survey Responses

**Table. Extended responses to the survey on knowledge and awareness of post-TB health and well-being among physicians in The Netherlands.**

|  | Total | Public health physician^*^ | Pulmo-nologist^†^ | Paediatrician | Infection specialist |
| --- | --- | --- | --- | --- | --- |
| Total | 40 | 10 | 25 | 4 | 1 |
| **Survey respondent characteristics** | | | | | |
| Setting | | | | | |
| Academic hospital | 6 | 0 | 2 | 3 | 1 |
| General hospital | 21 | 0 | 21 | 0 | 0 |
| Public health | 13 | 10 | 2 | 1 | 0 |
| Age range patient population | | | | | |
| All ages | 10 | 8 | 2 | 0 | 0 |
| <18 years | 5 | 0 | 1 | 4 | 0 |
| ≥18 years | 25 | 2 | 22 | 0 | 1 |
| **Knowledge and awareness of post-TB health and well-being** | | | | | |
| To what extent do you know about post-TB health and well-being | | | | | |
| None | 8 | 1 | 6 | 1 | 0 |
| Very little | 6 | 1 | 2 | 3 | 0 |
| Some | 20 | 5 | 14 | 0 | 1 |
| A lot | 6 | 2 | 3 | 0 | 0 |
| To what extent do you believe post-TB lung health and well-being is a problem among TB patients in The Netherlands? | | | | | |
| None | 2 | 0 | 2 | 0 | 0 |
| Very little | 13 | 6 | 6 | 1 | 0 |
| Some | 20 | 3 | 14 | 3 | 0 |
| A lot | 5 | 1 | 3 | 0 | 1 |
| To what extent do you believe post-TB lung disease is a problem among Dutch TB patients? | | | | | |
| None | 3 | 0 | 3 | 0 | 0 |
| Very little | 11 | 6 | 5 | 0 | 0 |
| Some | 24 | 4 | 16 | 4 | 0 |
| A lot | 2 | 0 | 1 | 0 | 1 |
| **Post-TB lung health in practice** | | | | | |
| Which examinations do you conduct at the completion of TB treatment for pulmonary TB? [Multiple answers] | | | | | |
| Concluding consult | 38 | 10 | 24 | 3 | 1 |
| CXR | 32 | 10 | 20 | 1 | 1 |
| Lung function test | 6 | 1 | 3 | 2 | 0 |
| 6 min walking test | 0 | 0 | 0 | 0 | 0 |
| Other^‡^ | 6 | 2 | 4 | 0 | 0 |
| Do you always follow up with TB survivors after the successful completion of TB treatment? | | | | | |
| Yes | 15 | 5 | 8 | 1 | 1 |
| No | 25 | 5 | 17 | 3 | 0 |
| Do you follow up with TB patients (on indication) after the conclusion of medical treatment | | | | | |
| Yes | 40 | 10 | 25 | 4 | 1 |
| No | 0 | 0 | 0 | 0 | 0 |
| Which examinations do you conduct on indication and at the completion of TB treatment for pulmonary TB? [Multiple answers] | | | | | |
| Concluding consult | 16 | 6 | 9 | 1 | 0 |
| CXR | 23 | 7 | 14 | 2 | 0 |
| Lung function test | 19 | 0 | 16 | 2 | 1 |
| 6 min walking test | 2 | 1 | 0 | 1 | 0 |
| Other^§^ | 16 | 6 | 9 | 1 | 0 |
| On what indication, how often and for how long have you provided follow-up care to patients? [Open question: summary of responses] | | | | | |
| Indication | Extrapulmonary manifestation; persistent health complaints; persisting cough complaints; persisting complaints of fatigue; functional deprivation; persisting abnormalities on CXR, including obstruction or fibrosis, bronchiectasis, pathologic bronchial arteriae; patients with extended pulmonary TB, including caverned; poor lung function; dyspnoea; malignancy | | | | |
| Frequency | Most physicians reported 1–3 patients per year; few physicians perform CXR after 6 months among all patients | | | | |
| Duration of follow-up | 3–6 months after treatment; up to 2 years; first 2 years every 6 months, after that annually for 5 years | | | | |
| What is the estimated proportion of patients you have treated and have followed up because of lung damage or post-TB lung disease? | | | | | |
| <10% | 19 | 5 | 11 | 3 | 0 |
| 10–20% | 12 | 2 | 9 | 0 | 1 |
| 20–30% | 4 | 2 | 2 | 0 | 0 |
| 30–40% | 4 | 1 | 2 | 1 | 0 |
| >40% | 1 | 0 | 1 | 0 | 0 |
| Do you feel there are unmet care needs among TB patients in the Netherlands? [Multiple answers] | | | | | |
| Yes,   - but those care needs are outside my profession - but there is currently no guideline - for those patients, I plan a follow-up appointment - I refer those patients to the GP or other specialist | 32  2  9  21  13 | 10  1  5  6  6 | 19  1  4  13  5 | 2  0  0  2  1 | 1  0  0  0  1 |
| No | 8 | 0 | 6 | 2 | 0 |
| What are the considerations or indications for planning a follow-up appointment with the patient? [Multiple answers] | | | | | |
| Exacerbated pulmonary TB | 32 | 9 | 20 | 2 | 1 |
| Disseminated TB | 23 | 7 | 14 | 1 | 1 |
| Persisting cough | 29 | 8 | 17 | 4 | 0 |
| Abnormal CXR | 29 | 9 | 17 | 2 | 1 |
| Abnormal lung function test | 21 | 6 | 11 | 3 | 1 |
| Other^¶^ | 6 | 2 | 4 | 0 | 0 |
| Do you think more research is warranted to assess the prevalence and severity of post-TB health and well-being and the type of interventions that should be applied in practice to improve post-TB health and well-being? | | | | | |
| Yes | 27 | 7 | 16 | 3 | 1 |
| No | 13 | 3 | 9 | 1 | 0 |
| Please indicate why research on post-TB is warranted [Open question: summary of responses] | | | | | |
| - To provide clarity, insight on the matter and guidance/impact on guidelines - Practical guidance that can be applied in practice - Guidance and tools are needed to identify persons at risk for post-TB sequelae - To understand the size and severity of the problem - Evidence on how post-TB sequelae emerge - Evidence on how post-TB sequelae can be prevented - Evidence on therapeutic interventions to treat post-TB sequelae - To provide better quality care | | | | | |
| What should be the focus of future research on post-TB and possible related interventions? | | | | | |
| - Overall QoL after successful completion of TB treatment - Health complaints affecting daily functioning in life - Primary pulmonary restricting complaints - Pulmonological conditions following TB, including COPD and bronchiectasis - Physical and mental health complaints post-TB treatment and unmet care needs - Psycho-social and economic consequences and long-term impact - Prevention - Role of physiotherapy and rehabilitation - Clinical management of complications - Best medical, endoscopic, and surgical treatment options - Best practices for monitoring and treating recidivism and post-TB | | | | | |

**^*^**One public health physician is also a pulmonologist, working in both public health and academic hospital settings (but classified as public health). ^†^One pulmonologist works both in general hospital and public health (classified as public health). ^‡^Additional laboratory (on indication), evaluation and follow-up, CT scan depending on CXR at start of treatment, CXR 1 year post-treatment, additional health education, and advice to contact the TB dept directly when complaints indicative of TB return. ^§^Exercise test, CT/MRI (depending on localisation), evaluation and follow-up actions, and other diagnostics depending on the situation and sequelae. ^¶^Specific forms of extrapulmonary TB, limited physical functioning, dyspnoea, MDR-TB, expected persisting health complaints or uncertainties, recidivism respiratory infection, pathological bronchial arteria.

CXR = chest X-ray; GP = general practitioner; COPD = chronic obstructive pulmonary disease; CT = computed tomography; MRI = magnetic resonance imaging; MDR-TB = multidrug-resistant TB.
